# Supplementary material for: Postinfection treatment with a protease inhibitor increases survival of mice with a fatal SARS-CoV-2 infection
Source: Proc Natl Acad Sci U S A. 2021 Jul 1;118(29):e2101555118. doi: 10.1073/pnas.2101555118 (PMC8307543; doi:10.1073/pnas.2101555118)
Supplement: Supplementary File [file pnas.2101555118.sapp.pdf]

Supplementary Information for

Post-infection treatment with a protease inhibitor increases survival of mice with a fatal SARS-CoV-2 infection

Chamandi S. Dampalla[1,7], Jian Zheng[2,7], Krishani Dinali Perera[3], Lok-Yin Roy Wong[2], David K. Meyerholz[4], Harry Nhat Nguyen[1], Maithri M. Kashipathy[5], Kevin P. Battaile[6], Scott Lovell[5], Yunjeong Kim[3*], Stanley Perlman[2*], William C. Groutas[1*], Kyeong-Ok Chang[3*]

[1]Department of Chemistry, Wichita State University, Wichita, Kansas 67260, USA

[2]Department of Microbiology and Immunology, University of Iowa, Iowa City, IA 52242, USA

[3]Department of Diagnostic Medicine and Pathobiology, College of Veterinary Medicine, Kansas State University, Manhattan, Kansas 66506, USA

[4]Department of Pathology, University of Iowa, Iowa City, IA 52242, USA

[5]Protein Structure Laboratory, The University of Kansas, Lawrence, Kansas 66047, USA

[6]NYX, New York Structural Biology Center, Upton, NY 11973, USA

[7]These authors contributed equally to this work.

* corresponding authors:

Kyeong-Ok Chang: kchang@vet.ksu.edu

Yunjeong Kim: ykim@ksu.edu

Stanley Perlman: Stanley-perlman@uiowa.edu

William C Groutas: bill.groutas@wichita.edu

This PDF file includes:

Supporting text

Figures S1 to S4

Table S1

SI References

Synthesis of deuterated 3CLpro inhibitors.

General. Reagents and dry solvents were purchased from various chemical suppliers (Sigma-Aldrich, Acros Organics, Chem-Impex, TCI America, Oakwood chemical, APExBIO, Cambridge Isotopes and Fisher) and were used as obtained. Silica gel (230-450 mesh) used for flash chromatography was purchased from Sorbent Technologies (Atlanta, GA). Thin layer chromatography was performed using Analtech silica gel plates. Visualization was accomplished using UV light and/or iodine. NMR spectra were recorded in $CDCl_3$ or DMSO-d_6 using a Varian XL-400 spectrometer. Melting points were recorded on a Mel-Temp apparatus and are uncorrected. High resolution mass spectrometry (HRMS) was performed at the University of Missouri-Kansas City Mass Spectroscopy Core facility, using an LCMS-9030 Q-TOF mass spectrometer equipped with Nexera X3 (40-series) UHPLC system, dual ion source (DUIS) and quadrupole time of flight (Q-ToF) mass analyzer (Shimadzu, Columbia, MD). Purity was >95% as evidenced by proton NMR analysis.

Compound *1*

Phenylmethyl-d2 ((S)-4-methyl-1-oxo-1-(((S)-1-oxo-3-((S)-2-oxopyrrolidin-3-yl)propan-2-yl)amino)pentan-2-yl)carbamate (***1***). Yield (57%), mp 45-48 ^{0}C. ^{1}H NMR (400 MHz, $CDCl_3$): δ 9.48 (s, 1H), 8.32 (d, J = 5.9 Hz, 1H), 7.38 – 7.28 (m, 5H), 6.18 (s, 1H), 5.44 (d, J = 8.6 Hz, 1H), 4.40 – 4.29 (m, 2H), 3.36 – 3.29 (m,

2H), 2.52 – 2.22 (m, 2H), 2.01 – 1.79 (m, 2H), 1.79 – 1.64 (m, 3H), 1.60 – 1.50 (m, 1H), 0.97 (d, *J* = 5.7 Hz, 6H). HRMS m/z: $[M+H]^+$ Calculated for $C_{21}H_{28}D_2N_3O_5$: 406.2311. Found: 406.2314; m/z: $[M+Na]^+$ Calculated for $C_{21}H_{28}D_2N_3NaO_5$: 428.2131. Found: 428.2136.

Compound *2*

Sodium (2S)-1-hydroxy-2-((S)-4-methyl-2-(((phenylmethoxy-d2)carbonyl)amino)pentanamido)-3-((S)-2-oxopyrrolidin-3-yl)propane-1-sulfonate (***2***). Yield (69%), mp 118-120 ^{0}C. ^{1}H NMR (400 MHz, DMSO-d_6) δ 7.65 (d, *J* = 9.2 Hz, 1H), 7.59 (d, *J* = 9.3 Hz, 1H), 7.49 (d, *J* = 8.4 Hz, 1H), 7.33 – 7.27 (m, 5H), 5.49 (d, *J* = 6.2 Hz, 1H), 5.33 (d, *J* = 6.0 Hz, 1H), 4.27 – 4.19 (m, 1H), 3.88 – 3.83 (m, 1H), 3.18j – 2.96 (m, 2H), 2.21 – 1.89 (m, 2H), 1.67 – 1.50 (m, 2H), 1.50 – 1.38 (m, 4H), 0.83 (d, *J* = 2.9 Hz, 6H). HRMS m/z: $[M+H]^+$ Calculated for $C_{21}H_{29}D_2N_3NaO_8S$: 510.1855, Found: 510.1838.

Compound *3*

Sodium (5S,8S)-5-isobutyl-3,6,11-trioxo-8-(((S)-2-oxopyrrolidin-3-yl)methyl)-1-phenyl-2,10-dioxa-4,7-diazadodecane-9-sulfonate-1,1-d2 (***3***). Yield (72%), mp 110-113 ^{0}C. ^{1}H NMR (400 MHz, DMSO-d_6) δ 7.70 – 7.62 (m, 2H), 7.49 (s, 1H), 7.41 – 7.30 (m, 5H), 5.10 (d, *J* = 8.9 Hz, 1H), 4.41 – 3.80 (m, 2H), 3.18 – 3.03 (m, 2H), 2.30 – 1.82 (m, 2H), 1.77 (s, 3H), 1.69 – 1.32 (m, 6H), 0.83 (d, *J* = 5.9 Hz, 6H). HRMS m/z: $[M+H]^+$ Calculated for $C_{23}H_{31}D_2N_3NaO_9S$: 552.1960. Found: 552.1953; m/z: $[M+Na]^+$ Calculated for $C_{23}H_{30}D_2N_3Na_2O_9S$: 574.1780, Found: 574.1777. m/z: $[M]^-$ Calculated for $C_{23}H_{30}D_2N_3O_9S$: 528.1984, Found: 528.1993.

Compound *4*

Sodium (5S,8S)-5-isobutyl-3,6,11-trioxo-8-(((S)-2-oxopyrrolidin-3-yl)methyl)-1-phenyl-2,10-dioxa-4,7-diazahexadecane-9-sulfonate-1,1-d2 (***4***). Yield (64%), mp 100-104 ^{0}C. ^{1}H NMR (400 MHz, DMSO-d_6) δ 7.68 (d, *J* = 10.4 Hz, 1H), 7.51 (d, *J*

= 8.2 Hz, 1H), 7.47 (s, 1H), 7.42 – 7.23 (m, 5H), 5.11 (d, *J* = 8.7 Hz, 1H), 4.34 – 4.00 (m, 1H), 4.00 – 3.83 (m, 1H), 3.21 – 2.91 (m, 2H), 2.41 – 1.99 (m, 4H), 1.99 – 1.74 (m, 1H), 1.74 – 1.33 (m, 7H), 1.35 – 1.16 (m, 4H), 0.86 (d, *J* = 12.6 Hz, 9H). HRMS m/z: $[M+H]^+$ Calculated for $C_{27}H_{39}D_2N_3NaO_9S$: 608.2586. Found: 608.2581; m/z: $[M+Na]^+$ Calculated for $C_{27}H_{38}D_2N_3Na_2O_9S$: 630.2406, Found: 630.2404; m/z: $[M]^-$ Calculated for $C_{27}H_{38}D_2N_3O_9S$: 584.2610, Found: 584.2623.

Compound *5*

Phenylmethyl-d2 ((S)-1-(((S)-4-(benzylamino)-3,4-dioxo-1-((S)-2-oxopyrrolidin-3-yl)butan-2-yl)amino)-4-methyl-1-oxopentan-2-yl)carbamate (***5***). Yield (40%), mp 124-128 ^{0}C. ^{1}H NMR (400 MHz, $CDCl_3$) δ 8.43 (d, *J* = 6.1 Hz, 1H), 7.49 (t, *J* = 6.2 Hz, 1H), 7.37 – 7.19 (m, 10H), 6.89 (s, 1H), 5.73 (d, *J* = 8.7 Hz, 1H), 4.48 – 4.42 (m, 2H), 4.42 – 4.17 (m, 2H), 3.32 – 3.09 (m, 2H), 2.57 – 2.43 (m, 1H), 2.43 – 2.29 (m, 1H), 2.29 – 2.12 (m, 1H), 1.96 – 1.81 (m, 1H), 1.81 – 1.56 (m, 3H), 1.56 – 1.45 (m, 1H), 0.93 (d, *J* = 6.5 Hz, 6H). HRMS m/z: $[M+H]^+$ Calculated for $C_{29}H_{35}D_2N_4O_6$: 539.2838, Found: 539.2842, m/z: $[M+Na]^+$ Calculated for $C_{29}H_{34}D_2N_4NaO_6$: 561.2658, Found: 561.2661.

Compound *6*

(Phenyl-d5)methyl ((S)-4-methyl-1-oxo-1-(((S)-1-oxo-3-((S)-2-oxopyrrolidin-3-yl)propan-2-yl)amino)pentan-2-yl)carbamate (***6***). Yield (62%), mp 40-43 ^{0}C. ^{1}H NMR (400 MHz, DMSO-d_6) δ 9.40 (s, 1H), 8.48 (d, *J* = 7.6 Hz, 1H), 7.49 (s, 1H), 7.38 (d, *J* = 8.3 Hz, 1H), 5.03 – 5.03 (m, 2H), 4.24 – 4.13 (m, 1H), 4.09 – 3.99 (m, 1H), 3.19 – 2.92 (m, 2H), 2.37 – 1.95 (m, 2H), 1.89 – 1.75 (m, 1H), 1.71 – 1.48 (m, 2H), 1.47 – 1.33 (m, 3H), 0.86 (d, *J* = 2.1 Hz, 6H). HRMS m/z: $[M+H]^+$ Calculated for $C_{21}H_{25}D_5N_3O_5$: 409.2499, Found: 409.2528, m/z: $[M+Na]^+$ Calculated for $C_{21}H_{24}D_5N_3NaO_5$: 431.2319, Found: 431.2348.

Compound *7*

Sodium (2S)-1-hydroxy-2-((S)-4-methyl-2-((((phenyl-d5)methoxy)carbonyl)amino)pentanamido)-3-((S)-2-oxopyrrolidin-3-yl)propane-1-sulfonate (***7***). Yield (68%), mp 125-128 ^{0}C. ^{1}H NMR (400 MHz, DMSO-d_6) δ 7.64 (d, *J* = 9.1 Hz, 1H), 7.59 (d, *J* = 9.3 Hz, 1H), 7.49 (d, *J* = 8.1 Hz, 1H), 5.44 (d, *J* = 6.3 Hz, 2H), 5.29 (d, *J* = 6.0 Hz, 1H), 5.05 – 4.98 (m, 2H), 4.03 – 3.87 (m, 2H), 3.17 – 2.95 (m, 2H), 2.24 – 2.02 (m, 2H), 1.83 – 1.50 (m, 3H), 1.49 – 1.37 (m, 2H), 0.84 (d, *J* = 3.0 Hz, 6H). HRMS m/z: $[M+H]^+$ Calculated for $C_{21}H_{26}D_5N_3NaO_8S$: 513.2043, Found: 513.2054, m/z: $[M+Na]^+$ Calculated for $C_{21}H_{25}D_5N_3Na_2O_8S$: 535.1863, Found: 535.1904.

Compound *8*

Sodium (5S,8S)-5-isobutyl-3,6,11-trioxo-8-(((S)-2-oxopyrrolidin-3-yl)methyl)-1-(phenyl-d5)-2,10-dioxa-4,7-diazahexadecane-9-sulfonate (***8***). Yield (67%), mp 110-112 ^{0}C. ^{1}H NMR (400 MHz, DMSO-d_6) δ 7.87 (d, *J* = 9.3 Hz, 1H), 7.68 (d, *J* = 9.9 Hz, 1H), 7.50 (d, *J* = 9.5 Hz, 1H), 5.11 (d, *J* = 8.9 Hz, 1H), 5.04 – 5.01 (m, 2H), 4.38 – 4.04 (m, 1H), 4.04 – 3.80 (m, 1H), 3.21 – 2.89 (m, 2H), 2.37 – 2.06 (m, 2H), 2.01 (t, *J* = 7.4 Hz, 3H), 1.98 – 1.67 (m, 0H), 1.67 – 1.55 (m, 4H), 1.45 (p, *J* = 7.4 Hz, 2H), 1.39 – 1.33 (m, 1H), 1.32 – 1.16 (m, 4H), 0.93 – 0.76 (m, 9H). HRMS m/z: $[M+H]^+$ Calculated for $C_{27}H_{36}D_5N_3NaO_9S$: 611.2775, Found: 611.2816.

Compound *9*

(Phenyl-d5)methyl-d2 ((S)-4-methyl-1-oxo-1-(((S)-1-oxo-3-((S)-2-oxopyrrolidin-3-yl)propan-2-yl)amino)pentan-2-yl)carbamate (***9***). Yield (70%), mp 45-48 ^{0}C. ^{1}H NMR (400 MHz, DMSO-d_6) δ 9.40 (s, 1H), 8.48 (d, *J* = 7.6 Hz, 1H), 7.63 (s, 1H), 7.47 (d, *J* = 8.3 Hz, 1H), 4.23 – 4.17 (m, 1H), 4.12 – 4.03 (m, 1H), 3.23 – 2.95 (m, 2H), 2.37 – 2.03 (m, 3H), 1.93 – 1.81 (m, 1H), 1.74 – 1.56 (m, 3H), 1.56 – 1.36 (m, 1H), 0.87 (d, *J* = 6.6 Hz, 6H). HRMS m/z: $[M+H]^+$ Calculated for $C_{21}H_{23}D_7N_3O_5$:

411.2625, Found: 411.2622, m/z: [M+Na]$^+$ Calculated for $C_{21}H_{22}D_7N_3NaO_5$: 433.2445, Found: 433.2440.

Compound *10*

Sodium (2S)-1-hydroxy-2-((S)-4-methyl-2-((((phenyl-d5)methoxy-d2)carbonyl)amino)pentanamido)-3-((S)-2-oxopyrrolidin-3-yl)propane-1-sulfonate (***10***). Yield (82%), mp 120-122 ^{0}C. ^{1}H NMR (400 MHz, DMSO-d_6) δ 7.66 (d, *J* = 9.1 Hz, 0H), 7.60 (d, *J* = 9.5 Hz, 1H), 7.50 (s, 1H), 5.51 (d, *J* = 6.2 Hz, 1H), 5.34 (d, *J* = 6.0 Hz, 0H), 4.06 – 3.97 (m, 1H), 3.97 – 3.90 (m, 1H), 3.15 – 3.07 (m, 2H), 2.21 – 2.01 (m, 3H), 2.01 – 1.71 (m, 1H), 1.71 – 1.49 (m, 4H), 1.49 – 1.31 (m, 2H), 0.87 (d, *J* = 4.7 Hz, 6H). HRMS m/z: [M+H]$^+$ Calculated for $C_{21}H_{24}D_7N_3NaO_8S$: 515.2169, Found: 515.2152.

Compound *11*

Sodium (5S,8S)-5-isobutyl-3,6,11-trioxo-8-(((S)-2-oxopyrrolidin-3-yl)methyl)-1-(phenyl-d5)-2,10-dioxa-4,7-diazahexadecane-9-sulfonate-1,1-d2 (***11***). Yield (44%), mp 100-105 ^{0}C. ^{1}H NMR (400 MHz, DMSO-d_6) δ 7.64 (d, *J* = 8.7 Hz, 1H), 7.48 (s, 1H), 7.35 (d, *J* = 8.0 Hz, 1H), 5.13 (d, *J* = 8.8 Hz, 1H), 4.16 – 4.03 (m, 1H), 4.05 – 3.87 (m, 1H), 3.21 – 2.87 (m, 2H), 2.40 – 1.99 (m, 4H), 1.99 – 1.76 (m, 1H), 1.76 – 1.34 (m, 7H), 1.34 – 1.18 (m, 4H), 0.96 – 0.69 (m, 9H). HRMS m/z: [M+H]$^+$ Calculated for $C_{27}H_{34}D_7N_3NaO_9S$: 613.2900, Found: 613.2899, m/z: [M]$^-$ Calculated for $C_{27}H_{33}D_7N_3O_9S$: 589.2924, Found: 589.2940.

Figure S1. Synthesis of deuterated inhibitors ***1-11***

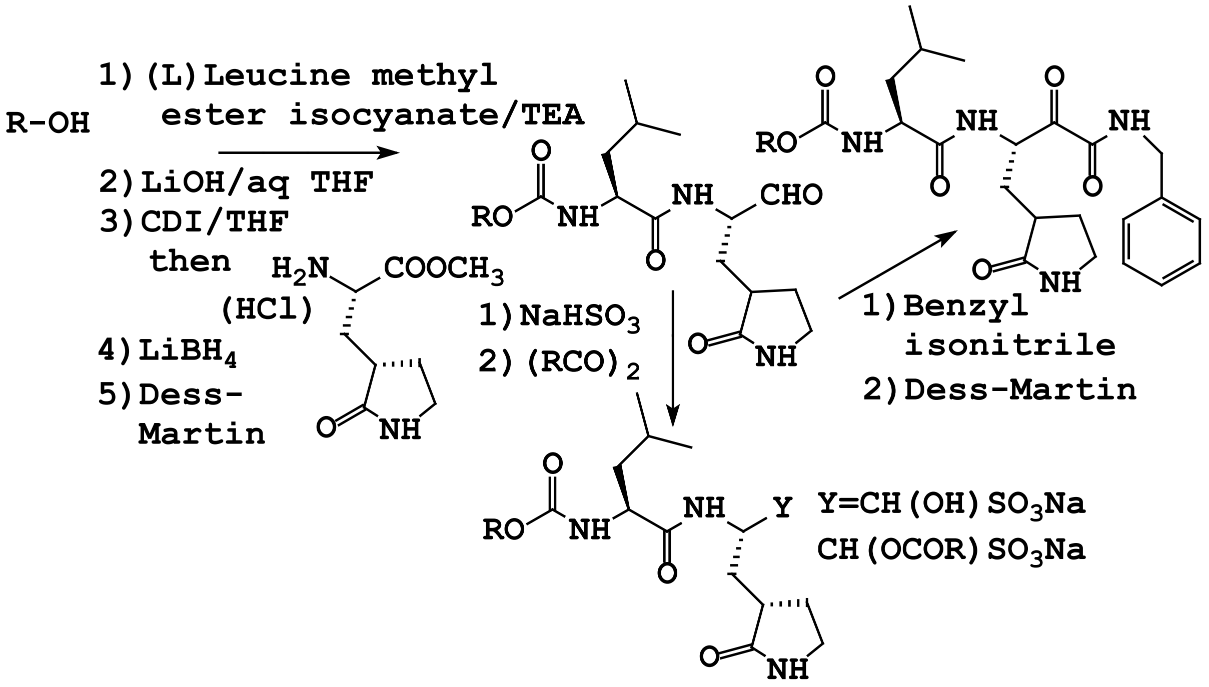

Figure S2. Structure of SARS-CoV-2 3CLpro in complex with compound ***2*** (gray) superimposed with (A) SARS-CoV-2 3CLpro with GC376 (PDB 6WTJ, gold) and *(*B) SARS-CoV-2 3CLpro with GC373 (PDB 6WTK, coral).

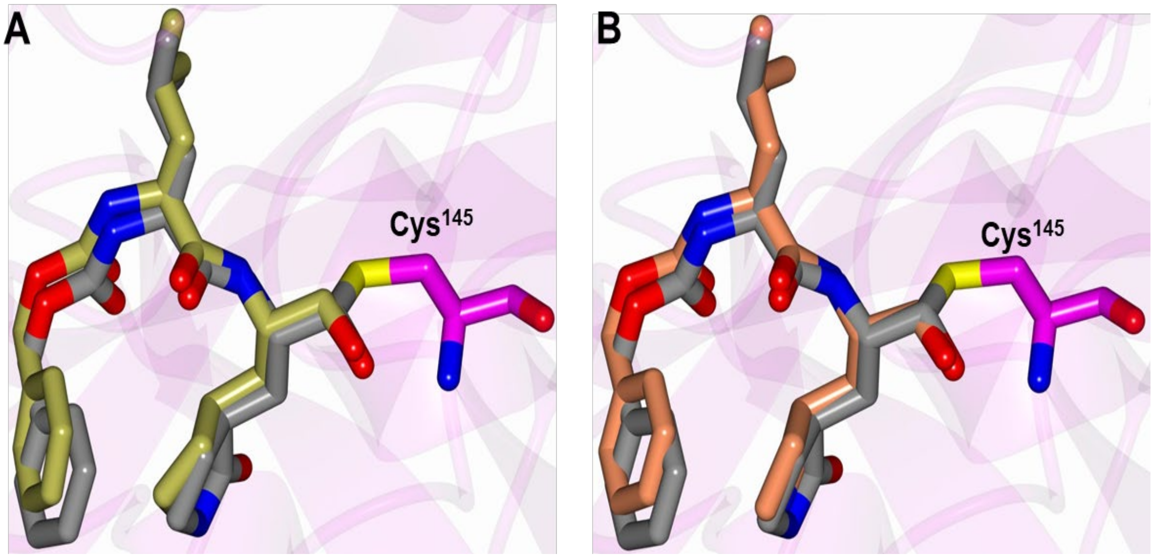

Figure S3. Cocrystal structures of SARS-CoV 3CLpro (A, B, C) and SARS-CoV-2 3CLpro (D, E, F) in complex with compound ***5***. Panels A and D show F_o-F_c omit maps (green mesh) contoured at 3σ. Panels B and E show hydrogen bond interactions (dashed lines) between the inhibitor and the 3CL protease. Panels C and F show electrostatic surface representation of the binding pocket occupied by the inhibitor. Neighboring residues are colored yellow (nonpolar), cyan (polar), and white (weakly polar).

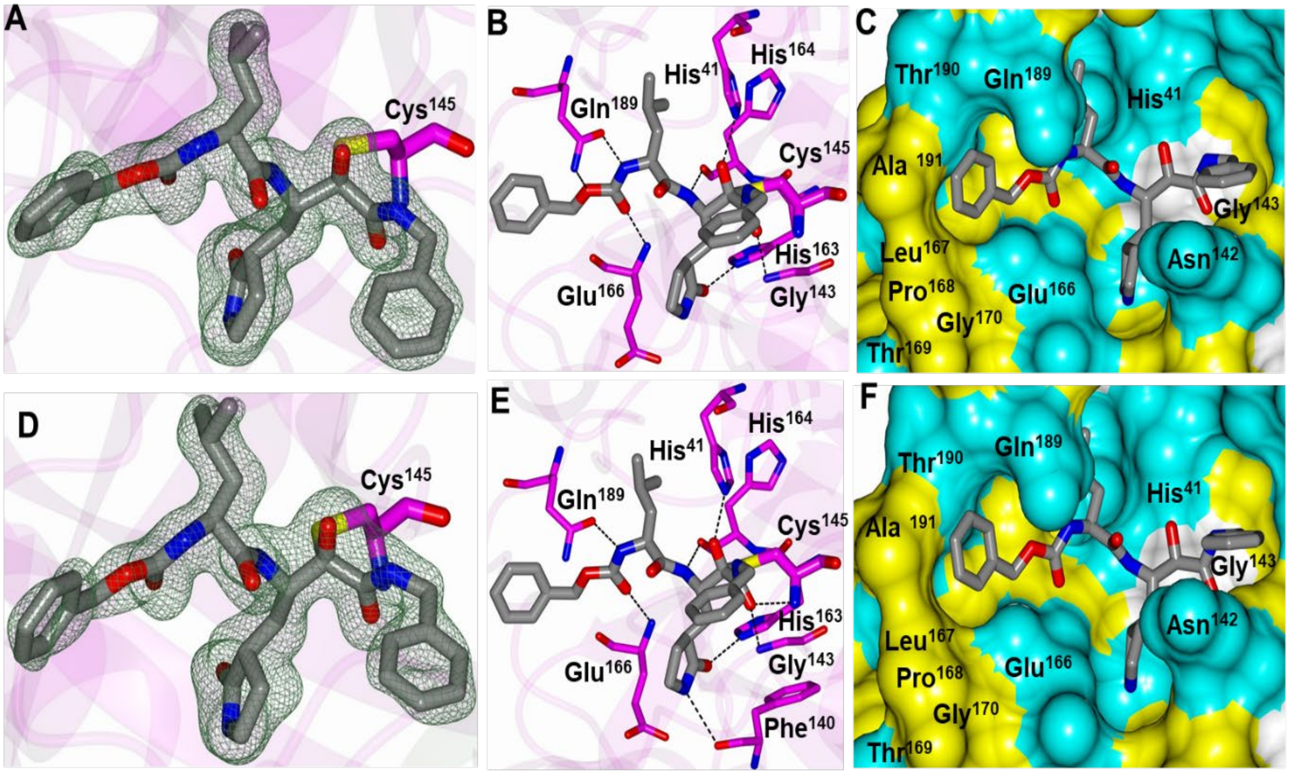

Figure S4. Structure of SARS-CoV-2 3CLpro in complex with compound ***5*** associated with subunit B. (A) Structure of compound **2** (gold) superimposed onto compound **5** in subunit B (gray) showing the similar binding modes. (B) F_o-F_c omit map (green mesh) contoured at 3σ. (C) Hydrogen bond interactions (dashed lines) between the inhibitor and the 3CL protease. (D) Electrostatic surface representation of the binding pocket occupied by the inhibitor. Neighboring residues are colored yellow (nonpolar), cyan (polar), and white (weakly polar).

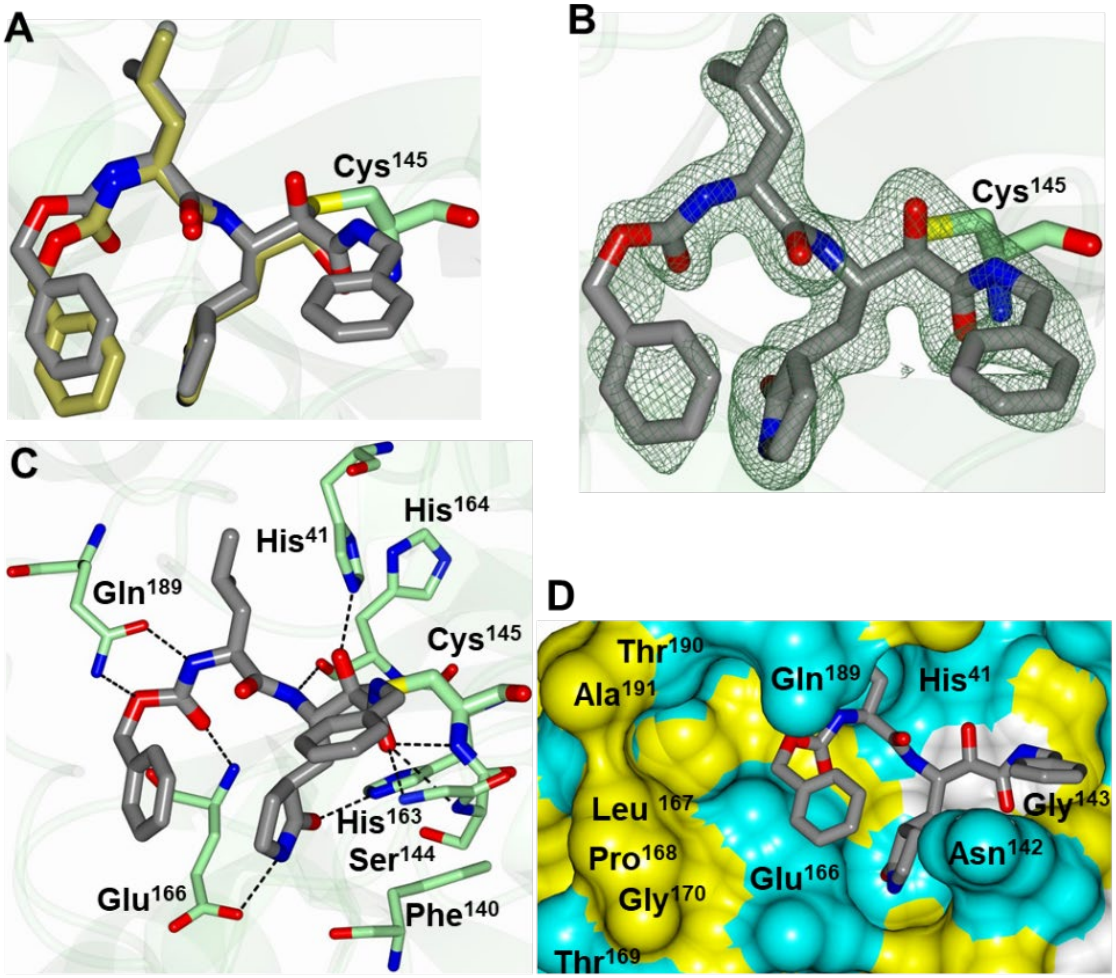

Table S1. Crystallographic data for SARS-CoV and SARS-CoV-2 3CLpro structures.

	**SARS-CoV-2 compound *2***	**SARS-CoV-2 compound *5***	**SARS-CoV compound *2***	**SARS-CoV compound *5***
**Data Collection**				
Unit-cell parameters (Å, °)	*a*=55.12 *b*=98.83 *c*=58.95 β=107.8	*a*=55.25 *b*=98.53 *c*=58.96 β=108.0	*a*=107.88 *b*=45.19 *c*=53.52	*a*=55.03 *b*=98.97 *c*=59.67 *b*=108.0
Space group	$P2_1$	$P2_1$	$P2_12_12$	$P2_1$
Resolution (Å)[1]	49.42-1.90 (1.94-1.90)	46.36-1.65 (1.68-1.65)	47.94-1.85 (1.89-1.85)	49.49-1.70 (1.73-1.70)
Wavelength (Å)	1.0000	0.9201	1.000	1.0000
Temperature (K)	100	100	100	100
Observed reflections	163,367	247,132	187,771	283,870
Unique reflections	46,687	70,687	23,106	66,700
$\langle I/\sigma(I) \rangle$[1]	8.8 (2.0)	10.7 (1.6)	13.7 (1.9)	10.5 (2.0)
Completeness (%)[1]	98.6 (100)	98.3 (96.9)	100 (100)	99.9 (100)
Multiplicity[1]	3.5 (3.6)	3.5 (3.5)	8.1 (8.4)	4.3 (4.4)
R_{merge} (%)[1, 2]	7.4 (56.5)	6.1 (79.0)	7.6 (120.5)	6.7 (75.1)
R_{meas} (%)[1, 4]	8.7 (66.4)	7.2 (93.3)	8.1 (128.4)	7.7 (85.5)
R_{pim} (%)[1, 4]	4.6 (34.6)	3.8 (49.1)	2.9 (43.8)	3.7 (40.3)
$CC_{1/2}$ [1, 5]	0.996 (0.845)	0.998 (0.676)	0.998 (0.679)	0.997 (0.822)
**Refinement**				
Resolution (Å) [1]	37.09-1.90	46.36-1.65	34.64-1.85	46.27-1.70
Reflections (working/test)[1]	44,269/2,348	67,180/3,463	21,933/1,130	63,330/3,304
R_{factor} / R_{free} (%)[1,3]	18.1/23.0	18.1/22.5	19.2/24.0	17.4/21.0
No. of atoms (Protein/Ligand/ Water)	4,483/58/197	4,493/78/337	2,203/58/127	4,510/72/298

**Model Quality**				
R.m.s deviations				
Bond lengths (Å)	0.011	0.011	0.009	0.010
Bond angles (°)	1.069	1.037	1.000	1.053
Mean *B*-factor (Å^2)				
All Atoms	37.4	29.6	40.1	32.5
Protein	37.3	29.0	40.1	32.0
Ligand	39.4	34.3	34.2	33.7
Water	40.1	36.0	42.5	38.4
Coordinate error	0.24	0.21	0.24	0.18
(maximum likelihood) (Å)				
Ramachandran Plot				
Most favored (%)	96.6	98.1	94.8	98.0
Additionally allowed (%)	3.4	1.9	4.5	2.0

1) Values in parenthesis are for the highest resolution shell.
2) *R*merge = Σ*hkl*Σ*i* |*Ii*(*hkl*) - <*I*(*hkl*)>| / Σ*hkl*Σ*i* *Ii*(*hkl*), where *Ii*(*hkl*) is the intensity measured for the *i*th reflection and <*I*(*hkl*)> is the average intensity of all reflections with indices hkl.
3) *R*factor = Σ*hkl* ||*F*obs (*hkl*) | - |*F*calc (*hkl*) || / Σ*hkl* |*F*obs (*hkl*)|; Rfree is calculated in an identical manner using 5% of randomly selected reflections that were not included in the refinement.
4) *R*meas = redundancy-independent (multiplicity-weighted) *R*merge(1, 2). *R*pim = precision-indicating (multiplicity-weighted) *R*merge(3, 4).
5) CC1/2 is the correlation coefficient of the mean intensities between two random half-sets of data(5, 6).

Supporting Information References

1. P. R. Evans, An introduction to data reduction: space-group determination, scaling and intensity statistics. *Acta Crystallogr D Biol Crystallogr* **67**, 282-292 (2011).
2. P. Evans, Scaling and assessment of data quality. *Acta Crystallogr D Biol Crystallogr* **62**, 72-82 (2006).
3. K. Diederichs, P. A. Karplus, Improved R-factors for diffraction data analysis in macromolecular crystallography. *Nat Struct Biol* **4**, 269-275 (1997).
4. M. S. Weiss, Global indicators of X-ray data quality. *J Appl Crystallogr* **34**, 130-135 (2001).
5. P. A. Karplus, K. Diederichs, Linking crystallographic model and data quality. *Science* **336**, 1030-1033 (2012).
6. P. Evans, Biochemistry. Resolving some old problems in protein crystallography. *Science* **336**, 986-987 (2012).
